# Molecular Imaging: Unveiling Metabolic Abnormalities in Pancreatic Cancer

**DOI:** 10.3390/ijms26115242

**Published:** 2025-05-29

**Authors:** Huanyu Wang, Yang Gui, Ke Lv

**Affiliations:** Department of Ultrasound, Peking Union Medical College Hospital, Peking Union Medical College, Chinese Academy of Medical Sciences, Beijing 100730, China; wanghuanyu@pumch.cn (H.W.); guiyang@pumch.cn (Y.G.)

**Keywords:** molecular imaging, metabolic imaging, pancreatic cancer

## Abstract

Pancreatic cancer remains one of the most aggressive malignancies globally, with a 5-year survival rate of less than 13%. This poor prognosis stems from late-stage diagnosis and intrinsic resistance to conventional therapies, including chemotherapy and radiotherapy. A hallmark of PC is oncogene-driven metabolic reprogramming—notably mediated by mutations in KRAS and other key pathways—which fuels tumor progression and undermines the efficacy of neoadjuvant treatments. Consequently, there is a pressing demand for non-invasive techniques capable of mapping metabolic alterations at both the tumor microenvironmental and systemic levels. This review will discuss molecular imaging techniques that identify metabolic changes within the tumor microenvironment. By bridging preclinical insights with clinical applications, we highlight how these innovations promise to revolutionize PC diagnosis, treatment stratification, and therapeutic monitoring, ultimately paving the way for precision oncology.

## 1. Introduction

Pancreatic cancer (PC) is one of the most aggressive and lethal malignancies worldwide, with a 5-year survival rate of less than 13% [1,2]. The majority of patients with this malignancy are diagnosed at an advanced stage, precluding eligibility for radical surgical resection, which is the only potentially curative treatment option at present [2]. Systematic chemotherapy regimens, including FOLFRINOX and gemcitabine in combination with nab-paclitaxel, continue to be the cornerstone of treatment for individuals with advanced disease [2]. Despite their utility, the rapid development of chemoresistance leads to poor prognosis [3].

A key driver of chemoresistance and tumor progression in PC is metabolic reprogramming, a hallmark of cancer that enables adaptation to the high energy and biosynthetic demands of rapidly proliferating cells [4,5]. PC cells rewire their metabolism to satisfy elevated energy requirements necessary for sustaining tumor progression in a nutrient-deprived and hypoxic tumor microenvironment (TME) [6]. This reprogramming encompasses alterations in the metabolism of glucose, lipids, and amino acids, in addition to dynamic interactions with stromal and immune cells within the TME [7].

The pathogenesis of pancreatic cancer is primarily driven by several critical mutations, notably including *KRAS*, which is present in more than 90% of cases [8]. This oncogene plays a central role in mediating metabolic reprogramming that fuels pancreatic carcinogenesis [9,10,11]. Recent advances in transcriptome profiling have enabled the molecular stratification of pancreatic adenocarcinoma (PDAC) into distinct subtypes [12]. These disparate classification schemes share fundamental commonalities. For example, the squamous (Bailey et al.), quasi-mesenchymal (Collisson et al.), and basal-like (Moffitt et al.) subtypes share many similarities at the transcriptomic level [12]. Clinically, this molecular heterogeneity correlates with prognostic divergence, where patients with basal-like subtypes exhibit significantly reduced overall survival compared to those with classical subtypes [13]. Functional validation using metastatic PC-derived patient organoids (PDOs) has unveiled subtype-specific metabolic dichotomies; basal-like PDOs demonstrate heightened oxidative phosphorylation (OXPHOS) activity and increased sensitivity to MCP1 inhibition relative to their classical counterparts [14]. In addition, PC can also be divided into different metabolic phenotypes according to its energy sources or gene expression related to metabolic processes [15,16,17]. Metabolic subtyping based on preferential energy utilization pathways reveals additional prognostic stratification. Specifically, glycolytically dominant tumors portend worse clinical outcomes than cholesterogenic phenotypes [17]. These multilayered classification frameworks—integrating molecular signatures and metabolic dependencies—provide a rationale for developing subtype-targeted therapies. The pharmacological exploitation of critical metabolic nodes represents a promising avenue for precision oncology in PC [18,19].

Molecular imaging techniques, capable of resolving spatial and temporal metabolic heterogeneity, offer unprecedented opportunities to decode the metabolic reprogramming of cancer at both molecular and cellular levels [20]. The most common molecular imaging modalities are positron emission tomography (PET) and magnetic resonance spectroscopy or imaging (MRS/MRI) [20]. PET employs radiolabeled metabolic analogs (e.g., ^18^F-fluorodeoxyglucose and ^18^F-FDG) to non-invasively quantify metabolic activity, with tracer uptake typically measured via standardized uptake value (SUV) [20,21]. In contrast, MRS/MRI exploits the spin polarization properties of atomic nuclei (notably ^1^H and ^13^C) to determine the biochemical composition and spatial distribution of metabolites [22]. While conventional ^1^H-MRS provides valuable metabolic information, its clinical utility is constrained by inherent sensitivity limitations arising from low metabolite concentrations and spectral peak overlap [23]. Hyperpolarized (HP)-^13^C-labeled probes have emerged as a breakthrough technology, amplifying signal intensity and enabling the investigation of dynamic metabolic processes in preclinical models [24]. Parallel innovations in deuterium metabolic imaging (DMI) utilize ^2^H-labeled substrates to achieve enhanced sensitivity for mapping specific metabolites [25]. Other techniques, including optical and near-infrared (NIR) imaging, while predominantly employed in small animal research due to limited tissue penetration depth, provide unparalleled spatiotemporal resolution for mechanistic investigations [22].

**Figure 1:**
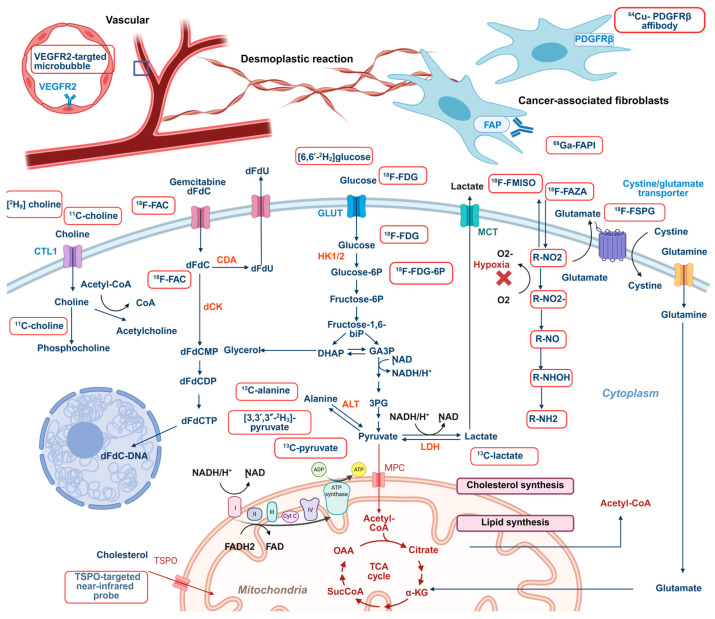
The landscape of key components in the metabolic imaging of PC. (Created in BioRender. Wang, A. (2025) https://BioRender.com/dekq39y). Key metabolites and targets for metabolic imaging and their metabolic processes are shown in the figure, with exogenous molecules highlighted in red boxes; 3-PG: 3-bisphosphoglycerate, ALT: alanine transaminase, CDA: cytidine deaminase, CoA: coenzyme A, CTL1: choline transporter like-protein 1, dCK: deoxycytidine kinase, DHAP: dihydroxyacetone phosphate, FAD: flavin adenine dinucleotide, fructose-6P: fructose 6-phosphate, fructose-1,6-biP: fructose 1,6-bisphosphate, R-NO2: NO2-containing molecular imaging probes, GA3P: glyceraldehyde 3-phosphate, GLUT1: glucose transporter 1, glucose-6P: glucose 6-phosphate, HK1/2: hexokinase 1/2, LDH: lactate dehydrogenase, MPC: mitochondrial pyruvate carrier, NAD: nicotinamide adenine dinucleotide, NADPH: nicotinamide adenine dinucleotide phosphate, OAA: oxaloacetate, TCA: tricarboxylic acid.

Despite these advancements, the clinical translation of metabolic imaging in PC remains limited. In this review, we systematically delineate key molecular imaging modalities and metabolic targets for use in characterizing tumor metabolism (Figure 1), with a focus on their emerging applications in guiding diagnosis, therapeutic stratification, and treatment monitoring in PC (Table 1). By synthesizing recent advances and unresolved challenges, we aim to bridge the gap between preclinical discoveries and clinical applications, thereby stimulating further research and innovation in this evolving field.

## 2. Molecular Imaging of Key Metabolic Pathways in Pancreatic Cancer: Glucose, Lipids, and Amino Acids

In this section, we focus on the major metabolic aberrations in glucose, lipid, and amino acid metabolism in PC and discuss how metabolic imaging can be utilized to identify these alterations.

### 2.1. Imaging Glucose Metabolism

The metabolic rewiring of pancreatic cancer cells toward aerobic glycolysis, first described by Otto Warburg in the 1920s, remains a defining hallmark of their malignant phenotype [26]. This metabolic reprogramming is characterized by the dramatic upregulation of glucose transporter 1 (GLUT1) and key rate-limiting glycolytic enzymes, including hexokinase 1/2 (HK1/2), phosphofructokinase 1 (PFK1), and lactate dehydrogenase (LDH). These molecular adaptations collectively enhance glycolytic flux, driving excessive lactate production, a hallmark of the Warburg effect [27,28,29].

**Figure 2:**
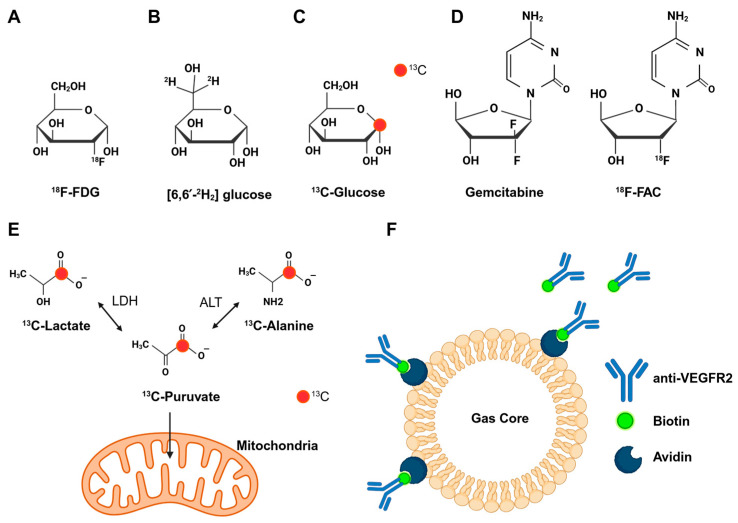
Probe structures or schematic diagrams of the probes for metabolic imaging. (Created in BioRender. Wang, A. (2025) https://BioRender.com/iihhl3v). (**A**) ^18^F-FDG; (**B**) [6,6-^2^H_2_] glucose; (**C**) ^13^C-Glucose; (**D**) gemcitabine and ^18^F-FAC; (**E**) the metabolism of ^13^C-Pyruvate in cancer; (**F**) schematic diagrams of VEGFR2-targeted microbubbles.

The profound glycolytic reprogramming in PC establishes key molecular targets for metabolic imaging. ^18^F-FDG PET (Figure 2A), often integrated with CT or MRI for anatomical correlation, has emerged as the cornerstone technique for visualizing glucose metabolism in vivo [30]. Following GLUT-mediated cellular uptake, FDG undergoes HK-dependent phosphorylation to FDG-6-phosphate, which is structurally incapable of proceeding through glycolysis due to C-2 fluorine substitution. While theoretically reversible via glucose-6-phosphatase (G6Pase)-mediated dephosphorylation, tumor-specific G6Pase deficiency creates a metabolic trap that promotes intracellular radiotracer retention, forming the biochemical basis of ^18^F-FDG PET signal quantification [31].

Clinically, ^18^F-FDG PET demonstrates diagnostic superiority over conventional anatomical imaging techniques and provides valuable prognostic information [32,33,34,35,36]. Dual-time ^18^F-FDG PET/CT offers quantitative insights into intra-tumoral metabolic heterogeneity, which has been associated with cancer treatment outcomes [37,38]. Yoo et al. demonstrated that a reduction in metabolic intra-tumoral heterogeneity during chemotherapy in patients with advanced-stage PC might serve as a predictor of progression-free survival (PFS) and overall survival (OS) [38]. Emerging evidence suggests that the multimodal integration of ^18^F-FDG PET/MRI with contrast-enhanced computed tomography (CECT) and CA19-9 monitoring enhances the accuracy of resectability prediction in patients undergoing neoadjuvant therapy, particularly for identifying vascular involvement [39]. In addition to ^18^F-FDG, other PET tracers are available for imaging glucose metabolism. Pyruvate kinase (PK) catalyzes pyruvate production, and its M2 splice variant, PKM2, has been shown to contribute to upregulated glycolysis in cancer cells [40]. ^18^F-DASA-23, a PKM2-specific isotopologue radiotracer, has demonstrated the ability to visualize PKM2 status in glioblastoma [41]. Although this tracer has yet to be tested in PC models, aberrant PKM2 expression and activation have been identified in PC and are associated with cancer cell survival and metastasis [42,43]. These findings provide a foundation for the potential future application of such tracers in pancreatic cancer research.

Pyruvate, the final product of glycolysis, serves as a pivotal junction in cellular metabolism, with three primary metabolic fates: (1) its conversion to acetyl-CoA via pyruvate dehydrogenase in the mitochondria, enabling entry into the tricarboxylic acid (TCA) cycle; (2) LDH-mediated lactate production; and (3) alanine aminotransferase (ALT)-catalyzed synthesis of alanine, supporting amino acid metabolism [44]. LDH catalyzes the conversion of pyruvate to lactate, and elevated LDH levels have been found to be associated with multiple types of tumors, including PC [45]. Metabolic crosstalk between stromal cells and PC cells results in the altered secretion and consumption of alanine [46]. Furthermore, distinct alanine-to-lactate ratios have been observed in experimental models of PC [47].

The emerging technique of HP-^13^C metabolic MRI complements conventional imaging by enabling the dynamic, real-time investigation of glycolytic flux. This technique holds promise for precursor lesion detection and the monitoring of chemotherapy response. In one study, the intravenous administration of HP-^13^C-labeled pyruvate enabled the non-invasive identification of PC precursor lesions through the quantification of ^13^C-alanine/^13^C-lactate signal ratios, which correlate with the alanine/lactate concentration ratio and also decrease with the ALT/LDH activity ratio during disease progression [23] (Figure 2E). In addition, in patients with locally advanced or metastatic PC, alanine-to-lactate ratios observed in HP-^13^C-labeled pyruvate MRI before and 4 weeks after treatment demonstrated altered metabolism following chemotherapy [47].

Furthermore, this technique aids in mapping metabolic heterogeneity. In patient-derived PC model systems, HP-^13^C-labeled pyruvate and lactate detected via MRS demonstrated functional metabolic heterogeneity in patient-derived primary cells, particularly when compared to conventional cell lines [48]. By detecting ^13^C-pyruvate and ^13^C-lactate interconversion in vivo, highly glycolytic PC xenografts could be identified non-invasively using HP-MRS [48]. Moreover, Kishmoto et al. demonstrated that, even with a similar genetic background, ^13^C-glucose tracers (Figure 2C) are able to distinguish PC xenografts by localizing lactate production and glucose metabolism [49]. Moreover, in another study, the combination of hyperpolarized MRI and ^18^F-FDG PET also provided quantitative insights into the relationship between glycolysis and oxygenation status inside tumors [30]. This approach revealed that tumor metabolism is heterogeneous, with ^18^F-FDG uptake negatively correlated with pO2 in the tumor core and positively correlated in the periphery. Unlike pO2 and ^18^F-FDG uptake, LDH activity was relatively evenly distributed throughout the tumor [30]. In another study, real-time metabolic imaging data also showed that the overexpression of LDH-A and hypoxia-inducible factor (HIF-1α) significantly influenced the conversion kinetics of HP pyruvate-to-lactate in PDX tumors [50].

Deuterium MRI (^2^H-MRI) was also applied in probing tumor glucose metabolism [51]. Through alternating deuterated and non-deuterated glucose injections, it was observed that tumors maintain relentless glucose consumption and lactate production whenever glucose is available. Lactate output appeared to be constrained solely by glucose availability, vividly illustrating the significant glucose consumption driven by the Warburg effect [51].

The pentose phosphate pathway (PPP) is also enhanced in PC [7]. During radiotherapy, the metabolic flux of PPP increases, with it being recognized that combining radiotherapy with PPP inhibitors can synergistically suppress tumor growth [52]. Research results from glioblastoma studies have demonstrated that after administering [2-^13^C]-glucose, ^13^C MRI can visualize the flux of [4-^13^C]-glutamate (produced via PPP) and [5-^13^C]-glutamate (generated through glycolysis), thereby reflecting changes in PPP activity within cells [53]. In light of the above findings, in PC, ^13^C MRI may serve as a potential tool for monitoring treatment response and tumor recurrence during radiotherapy.

### 2.2. Imaging Amino Acid Metabolism

Amino acid metabolism also undergoes profound transformation in PC, impacting tumorigenesis and development by influencing energy metabolism and signal transduction [54]. As the most abundant amino acid in blood plasma, glutamine (Gln) is involved in numerous important cellular processes, such as glucogenesis and the biosynthesis of proteins, and it also participates in the tricarboxylic acid (TCA) cycle [55,56,57]. Unlike most cells that utilize glutamate dehydrogenase (GLUD1) to convert glutamine-derived glutamate into α-ketoglutarate in the mitochondria for the TCA cycle, PC employs a unique cytoplasmic pathway [58]. In this process, glutamine-derived aspartate is metabolized via aspartate transaminase (GOT1) into oxaloacetate, malate, and then pyruvate, thereby enhancing the NADPH/NADP^+^ ratio to maintain redox balance [58]. In PC, Gln demand is increased, and glutamate ammonia ligase (GLUL), which is responsible for de novo synthesis, is upregulated in PC patient samples and mouse PC models [59].

Research indicates that single-voxel MRS could be used as a technique to measure metabolites, including Glx (glutamine and glutamate) and N-acetylaspartate (NAA) [60]. In one study, the levels of the aforementioned metabolites significantly decreased in PC when compared with a healthy pancreas [60]. Moreover, low Glx levels were found to be associated with poor PFS and OS [60]. (4S)-4-(3-18F-Fluoropropyl)-l-glutamate (FSPG) is a PET tracer designed to image the activity of a heterodimeric transporter that mediates the cellular uptake of cystine for the exchange of intracellular glutamine [61]. Compared to ^18^F-FDG PET, FSPG PET demonstrated higher sensitivity (95.0% vs. 90.0%), specificity (100.0% vs. 66.7%), and diagnostic accuracy (95.7% vs. 90.0%) in the detection of metastasis, particularly in the liver [61].

### 2.3. Imaging Lipid Metabolism

PC also demonstrates distinctive adaptive modifications in lipid metabolism that facilitate tumor progression, characterized by dual activation of exogenous uptake and dysregulated endogenous synthesis pathways [62]. Tumor cells acquire fatty acids (FAs) and cholesterol through multiple transporters. FA internalization is mediated by fatty acid transport proteins (FATPs), fatty acid translocase (CD36), and fatty acid-binding proteins (FABPs); in comparison, cholesterol is primarily acquired via low-density lipoprotein receptor (LDLR)-mediated endocytosis [63]. In head and neck squamous cell carcinoma (HNSCC), it has been reported that immunohistochemical (IHC) detection of CD36 expression makes it possible to differentiate false-negative lymph nodes in ^18^F-FDG-PET/CT imaging [64]. In a glioma orthotopic tumor model, a CD36-targeting NIR fluorescent probe was developed to visualize the CD36 protein and delineate tumor boundaries, expanding the toolbox for surgical navigation [65]. These studies collectively suggest a promising strategy for imaging lipid metabolism in PC.

De novo FA and cholesterol synthesis both rely on acetyl-CoA derived from glucose and glutamine metabolism [62]. Cholesterol imaging in vivo has been performed using PET probes [66,67]. However, such studies have yet to be conducted in pancreatic tumors. Cholesterol dysregulation in PC can be accessed through translocator protein (TSPO) imaging. In one study, TSPO, involved in cholesterol transport, showed elevated expression in high-grade pancreatic precancerous lesions [68]. In genetically engineered mouse models, a TSPO-targeted NIR probe was found to localize to pre-malignant pancreatic lesions and advanced-stage tumors, enabling real-time image-guided surgery [68].

Aberrant choline metabolism can also be identified through molecular imaging in PC. The use of ^1^H-MRSI resulted in the detection of increased levels of total choline in PC cell lines and tumors, corresponding to the elevated expression of choline kinase-α (Chkα), and the choline transporters CHT1 and CTL1 [69]. The authors of another study proposed that choline levels detected through the use of MRS were significantly higher in TNM stage 3+ PC tumors [70]. Other researchers have employed deuterium metabolic imaging (DMI) with the use of [^2^H_9_] choline and [6,6′-^2^H_2_] glucose (Figure 2B) to assess the uptake and conversion of choline and glucose [71]. The reconstruction of DMI data revealed the spatial accumulation patterns of ^2^H-labeled compounds, providing a more precise metabolic characterization of PC. Other choline-based tracers have also been synthesized for pre-clinical and clinical use, including 11C-choline, 18F-fluoro-choline (FCH), and 18F-fluoroethyl-choline (FEC) [72,73,74,75]. In a patient with prostate cancer, an 18F-FCH PET/CT scan revealed chronic mass-forming pancreatitis during evaluation for lymph node recurrence [76]. In another study, 11C-choline accumulation was observed in a metastatic pancreatic tumor originating from renal cell carcinoma; in comparison, 18F-FDG did not demonstrate similar uptake [77].

In human-derived PC cell lines, elevated levels of phosphocholine and phosphoethanolamine can also be detected [78]. To perform a comprehensive analysis of phosphorus metabolites, ^31^P chemical shift imaging is a commonly employed method [79]. Phosphorus metabolic imaging combined with 7T MR makes it possible to detect increased levels of phospholipid metabolites in tumor tissues compared to background signals, enabling non-invasive pH measurements and indicating the presence of hypoxia [80].

## 3. Imaging Phenotypic Consequences of Cancer Metabolism and Microenvironment

In this section, we will highlight imaging modalities designed to target key phenotypic traits of cancer metabolism, including altered redox homeostasis, chemotherapy drug metabolism, and tumor microenvironment changes such as hypoxia, desmoplasia, and vascularization.

### 3.1. Imaging Redox Homeostasis

While molecular oxygen (O_2_) serves as an indispensable substrate for ATP generation through OXPHOS in eukaryotic organisms, its metabolic processing paradoxically generates reactive oxygen species (ROS) through electron leakage. These redox-active molecules induce significant cellular damage, particularly within tumors. Therein, they induce genomic instability, promote malignant progression and metastasis, and confer resistance to chemotherapy [81]. The generation and detoxification of ROS are regulated by cellular metabolism [82,83]. The production of ROS is intricately linked with the activity of nicotinamide dinucleotide oxidases and flavin adenine dinucleotide (FAD) [84]. Nicotinamide dinucleotide (NADPH) and FAD are cycled between oxidized and reduced states to transfer electrons in the mitochondria through active TCA cycle reactions and OXPHOS [85].

Capitalizing on the inherent fluorescent properties of reduced NADPH and oxidized FAD, advanced imaging modalities enable the non-invasive monitoring of cellular metabolism [86,87]. Two-photon and multi-photon excitation microscopy techniques have been successfully implemented to quantify these autofluorescence signals in three-dimensional PC models, achieving subcellular resolution in intact tumor architectures [86,87]. In two studies, NADPH and FAD autofluorescence signals were measured at excitation and emission wavelengths of 400–480 nm and 500–600 nm, respectively [86,87]. In three-dimensional PC models, the data were integrated over a z-stack of 30 images acquired with 0.1 µm intervals. The optical redox ratio (ORR), defined as the ratio of NADPH-to-FAD fluorescence intensities, provides critical insights into cellular redox homeostasis by reflecting the relative abundance of electron donors versus acceptors. In addition, the fluorescence lifetimes of NADPH and FAD depend on their binding states to enzyme complexes, offering further metabolic insights. The composite optical metabolic imaging (OMI) index, integrating both ORR and lifetime parameters, has emerged as a robust quantitative biomarker for tracking metabolic reprogramming and therapeutic response in PC malignancies [86,87]. Recent applications in tumor organoid models have revealed critical metabolic crosstalk within the pancreatic tumor microenvironment. In their study, Datta et al. employed optical redox imaging in co-culture systems containing both neoplastic cells and stromal components, demonstrating that direct contact with pancreatic stellate cells (PSCs) significantly enhances oxidative metabolism in PC cells [88]. In complementary studies, researchers found that cancer-associated fibroblasts (CAFs) can induce redox state elevation in PC cells, correlating with acquired treatment resistance across both in vitro and in vivo models [89]. These findings underscore the importance of redox imaging as a powerful tool for elucidating tumor metabolism and its implications for therapeutic outcomes in PC.

### 3.2. Imaging the Metabolism of Chemotherapy Drugs

Gemcitabine, also known as 2′,2′-difluorodeoxycytidine (dFdC), is regarded as one of the first-line chemotherapy drugs for PC [2,3]. As a deoxycytidine nucleoside analog, gemcitabine effectively inhibits DNA synthesis, thereby arresting the cell cycle and suppressing cancer cell proliferation [90]. Gemcitabine relies on nucleoside transporters for cellular uptake and requires phosphorylation by deoxycytidine kinase (dCK) to exert its cytotoxic effects [3]. ^18^F-FAC (2′-deoxy-2′-18F-fluoro-β-d-arabinofuranosylcytosine), a PET imaging agent structurally analogous to gemcitabine, can also serve as a substrate for dCK [91] (Figure 2D). When integrated with MR, ^18^F-FAC PET/MR enabled the assessment of gemcitabine uptake in PDX models for PC. Notably, treatment with PEGylated recombinant human hyaluronidase (PEGPH20) was found to enhance tumor ^18^F-FAC uptake by 12% in these models [91].

In addition to PET imaging, the metabolism of gemcitabine can be visualized using mass spectrometry imaging (MSI), a label-free technique that provides high-resolution mapping of chemotherapy drugs and their metabolites in fresh frozen tissue sections [92]. In the LSL-Kras^G12D/+^, LSL-Trp53^R172H/+^, and Pdx-1-Cre (KPC) mouse models of PC, MSI has been successfully employed to map the spatial distribution of gemcitabine and its phosphorylated metabolites, including dFdCMP, dFdCDP, and dFdCTP, in addition to the inactive metabolite dFdU [92]. The biologically active phosphorylated metabolites were found to be related to the therapeutic function of gemcitabine [93]. This approach offers valuable insights into the pharmacokinetics and therapeutic response of gemcitabine in preclinical models.

### 3.3. Imaging Tumor Microenvironment: Hypoxia, Desmoplasia, and Vascularization

The complex TME, characterized by hypoxia and desmoplasia, interacts with tumor metabolism and plays a crucial role in the malignant progression of PC [94,95]. This interaction between the TME and tumor metabolism underscores the importance of understanding the interplay between these factors in disease progression. Molecular imaging offers a powerful tool to explore the TME by targeting key features such as hypoxia, desmoplasia, and vascularization. One approach to imaging hypoxia involves the use of nitroimidazole-based agents, such as ^18^F-FMISO and ^18^F-FAZA [96,97]. These agents, when employed in PET/CT imaging, provided valuable prognostic information for PC patients and aid in identifying suitable candidates for hypoxia-targeted therapy trials [96,97]. Furthermore, in orthotopic xenograft models of PC, dual-modality ultrasound (US) and photoacoustic (PA) imaging techniques have demonstrated the ability to measure relative tissue oxygenation [98]. This US/PA dual-modality imaging technique facilitates the acquisition of both anatomical and functional data, enhancing our understanding of tumor growth dynamics.

One hallmark of PC is the extensive desmoplastic reaction, which is primarily driven by the activation of CAFs [99]. Advancements in single-cell RNA sequencing (scRNAseq) have revealed the heterogeneity of CAF subpopulations, providing deeper insights into their roles in tumor progression [100]. Furthermore, the spatial distribution and temporal dynamics of CAFs underscore the importance of imaging techniques in understanding their contributions to tumor biology [101]. Fibroblast activation protein (FAP), which is highly and selectively expressed on CAFs, serves as a key imaging target [101]. To target FAP, various FAP inhibitor (FAPI) tracers have been designed and labeled with radionuclides including Gallium-68 (Ga68) and 18F for both diagnostic imaging and therapeutic applications [102,103]. There are currently numerous clinical studies underway to explore in greater detail the potential of these imaging techniques in PC diagnosis and treatment. The association between ^68^Ga-FAPI PET SUVmax and FAP expression, as determined using IHC, has been confirmed [104,105]. The results of a number of studies have demonstrated that ^68^Ga-FAPI PET results in superior or equivalent detection rates and diagnostic accuracy compared to ^18^F-FDG PET-CT [104,105,106,107,108,109]. In addition, the results of some studies suggest that ^68^Ga-FAPI-04 PET may outperform ^18^F-FDG PET in detecting suspicious lymph node metastases [110]. In terms of therapeutic management, ^68^Ga-FAPI PET-CT demonstrated a superior ability to identify distant metastases, particularly in advanced or recurrent cases, resulting in TNM stage reclassification when compared to CECT, which may lead to major treatment alterations [111,112]. Furthermore, in patients with PC following first-line treatment, a higher target-to-background ratio (TBR-blood) was associated with poor treatment response [113]. Collectively, these advances position FAPI-based imaging as a transformative tool for precision oncology in PC, enabling the non-invasive investigation of stromal biology and personalized therapeutic strategies. Moreover, CAFs have also been reported to be targeted by platelet-derived growth factor receptor beta (PDGFRβ) [114]. Following radiolabeling with ^64^Cu, PDGFRβ affibody ZPDGFRβ exhibited ideal imaging capabilities when combined with PET/CT in mouse models of PC [114].

The vascularization of PC can be effectively visualized using contrast-enhanced ultrasound (CEUS) with vascular endothelial growth factor receptor type 2 (VEGFR2)-targeted microbubbles [115] (Figure 2F). In transgenic mice with PC, VEGFR2-targeted ultrasound demonstrated significantly higher signal intensities in PC compared to the pancreas of wild-type mice, particularly in tumors smaller than 3 mm [115]. The above results highlight the potential of VEGFR2-targeted microbubbles for the earlier detection of small PC lesions [115]. Furthermore, clinical trials on the VEGFR2-targeted microbubble BR55 have been conducted in breast and ovarian lesions, demonstrating more pronounced targeted signals in malignant lesions compared to benign ones, thereby laying a solid foundation for its clinical application in oncology [116]. After being combined with the NIR probe IRDye800CW, the VEGFR2 monoclonal antibody bevacizumab could also be used for surgery guidance [117].

## 4. The Role of Artificial Intelligence (AI) in Enhancing Metabolic Imaging Interpretation

AI has undergone remarkable evolution in medical imaging, fundamentally transforming disease diagnosis and clinical management [118]. In PC research, significant efforts have focused on leveraging AI for predictive modeling, risk stratification, and diagnostic applications [119]. In the field of AI-assisted PC image analysis, key research directions include tumor growth modeling, organ/multi-organ segmentation and edge detection, pancreatic cancer diagnosis and risk assessment, prognostic prediction, and image and annotation quality optimization [119,120]. Cao et al.’s landmark study introduced PANDA, a deep learning system outperforming conventional non-contrast CT in pancreatic lesion detection and classification accuracy [121]. Placido et al. developed a machine learning model utilizing clinical history data for the risk prediction of PC [122]. Moreover, a 3D Contrast-Enhanced Convolutional Long Short-Term Memory network (CE-ConvLSTM) using CECT data was developed by researchers to evaluate tumor–vascular relationships, enabling survival prediction in patients with primarily resectable PC [123]. As discussed above, metabolic imaging modalities provide critical insights into the unique metabolic profile of pancreatic malignancies, offering essential information for both diagnosis and therapeutic planning. The integration of AI with metabolic imaging technologies presents a transformative opportunity to enhance diagnostic precision, enable earlier tumor detection, and potentially reveal novel metabolic biomarkers. A machine learning model was developed by researchers based on 18F-FDG PET/CT data combined with clinical features to predict tumor grade and prognosis in pancreatic neuroendocrine tumor patients [124]. Radiomics and the deep learning features of 18F-FDG PET/CT were also used to distinguish PC and autoimmune pancreatitis with an accuracy of 90.1% (95% CI: 88.7–91.5%) [125]. A deep learning model based on 18F-FDG-PET/CT examination before surgery was also developed by another group of researchers for pathological grading. Following integration with clinical data, the AUC of the model reached 0.77 [126]. Current investigative paradigms predominantly utilize PET/CT-based models that synergize radiological signatures with clinical parameters for pancreatic lesion classification and prognosis prediction. The future integration of AI with diverse metabolic imaging modalities and multi-omics data, including genomic sequencing in addition to spatial transcriptomics, holds significant potential for refining metabolic subtyping in PC.

AI applications in pancreatic cancer research currently face multiple challenges, with insufficient sample size being a particularly prominent issue. This limitation is especially acute in metabolic imaging—the inherent difficulties in data acquisition hinder the accumulation of adequately sized datasets, creating substantial barriers to reliable model training and validation, which ultimately results in unstable algorithmic performance. Moreover, the intrinsic ‘black-box’ nature of deep learning models and their lack of interpretability significantly impede the clinical translation and further development of these technologies [127].

## 5. Discussion and Conclusions

In conclusion, metabolic imaging has emerged as a revolutionary tool in PC research, significantly contributing to the elucidation of tumor metabolic abnormalities and offering critical insights into the TME.

**Figure 3:**
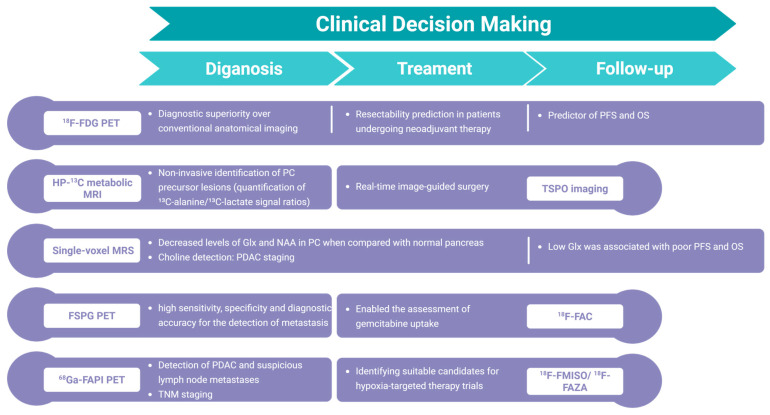
Potential role of metabolic imaging in clinical decision-making. (Created in BioRender. Wang, A. (2025) https://BioRender.com/pcrjp4k).

Emerging evidence from molecular imaging studies demonstrates that tumor metabolic profiling plays potential roles across the pancreatic cancer management spectrum, spanning early detection and precise staging to therapeutic monitoring and prognostic stratification (Figure 3). These advancements pave the way for novel therapeutic approaches tailored to individual patient needs. In PC management, real-time treatment monitoring is critically important. Although existing studies have established correlative associations between metabolic imaging findings and therapeutic response [112,128], there exists a paucity of direct evidence demonstrating how real-time metabolic changes detected through molecular imaging during treatment can reliably predict clinical outcomes.

Notably, in this review, we focus on established clinical applications from existing studies, though some potential uses remain unexplored. The clinical utility of metabolic imaging depends on both biological targets and imaging modalities. For example, PET/CT and PET/MRI are well suited for detecting distant metastases due to their whole-body scanning capability. NIR imaging offers real-time intraoperative navigation, making it ideal for surgical guidance. US/CEUS provides a non-invasive, radiation-free, and cost-effective approach, rendering it highly suitable for longitudinal treatment monitoring. The same molecular target can address different clinical needs when paired with appropriate imaging techniques. For instance, TSPO-targeted NIR probes assist in real-time surgery, whereas 18F-labeled TSPO tracers can aid in the early identification of early-stage pancreatic cancer lesions [68].

Metabolic imaging can also be combined with conventional serological biomarkers (e.g., CA19-9) to enhance diagnostic performance. Although they operate through fundamentally distinct mechanisms—metabolic imaging visualizes in vivo metabolic activity, whereas CA19-9 detects circulating tumor-derived carbohydrate antigens—their combined use enhances diagnostic confidence. When results are concordant, this integrated approach significantly strengthens either the confirmation or exclusion of PC. Combining CECT, PET, and CA19-9 may improve the assessment of resectability and recurrence prediction [39,129]. Furthermore, metabolic adaptations in PC significantly influence the levels of CA19-9. An integrated analysis of clinical and proteomic data from 128 resectable PC cases revealed that tumors with elevated CA19-9 levels exhibit enhanced glycolytic activity and demonstrate significant metabolic heterogeneity across multiple pathways—including carbohydrate, protein, amino acid, lipid, and nucleic acid metabolism—when compared to low-CA19-9-level tumors [130].

Beyond targeting metabolic products, processes, and phenotypic consequences as discussed herein, the imaging of upstream genetic mutations can also indicate potential metabolic abnormalities in tumors. For instance, KRAS plays a pivotal role in metabolic reprogramming in PC. Through the coordinated regulation of hexosamine, PPP, and glutamine pathways, oncogenic KRAS orchestrates a metabolic network supporting biosynthesis, redox homeostasis, and tumor proliferation [131]. The structural alterations induced by the G12C mutation enable inhibitor molecules to bind the allosteric switch II pocket (S-IIP) [132]. These small-molecule inhibitors can be conjugated with fluorescent probes (e.g., Alexa Fluor 647) via click chemistry, enabling the specific fluorescence imaging of KRAS G12C [133].

In addition to target identification and analytical methodologies, innovations in molecular probes and imaging materials also play important roles in the advancement of metabolic imaging. Nanotechnology-derived nanoparticles (NPs) are emerging as a transformative platform that may revolutionize molecular imaging through enhanced imaging performance and integrated diagnostic–therapeutic capabilities. A representative example is radiolabeled iron oxide (IO) nanoparticles, which enable the synergistic combination of high-sensitivity radionuclide imaging (SPECT/PET) with MRI, which offers outstanding spatial resolution [134]. CAF-targeted, controlled-release nanodroplets FH-V9302-siGLUL-NDs have also been developed to deliver the amino acid transporter ASCT2 (SLC1A5) inhibitor (V9302) and siRNA for *GLUL* (siGLUL) [128]. In one instance, FH-V9302-siGLUL-NDs serve as a CEUS imaging agent for tumor visualization. Conversely, through ultrasound-targeted microbubble destruction (UTMD), they enable the spatially controlled co-delivery of V9302 and siGLUL to disrupt the glutamine crosstalk between CAFs and tumor cells [128].

Together, advancements in functional imaging modalities and exploratory target selection are set to significantly enhance the identification of early-stage PC and the assessment of treatment responses, effectively tackling critical clinical challenges in disease management.

## Figures and Tables

**Table 1:** Key molecular imaging modalities in pancreatic cancer related to metabolism.

Imaging Technique	Tracers	Targeted Molecules or Processes	Applications	Stage ^1^
PET	^18^F-FDG	Glucose uptake	Diagnosis, staging, recurrence detection, resectability prediction, prognosis	Licensed tracers used in the clinic
	^18^F-FSPG	Cystine/glutamate transporter	Detection of metastasis	Clinical evaluation
	^18^F-FAC	dCK activity	Gemcitabine metabolism	Experimental modalities tested in animals
	^68^Ga-FAPI	FAP	Stromal mapping, metastasis detection, therapy response	Clinical evaluation
	64Cu- ZPDGFRβ	PDGFRβ	NA	Experimental modalities tested in animals
	^18^F-V-1008	TSPO	Distinguish early disease	Experimental modalities tested in animals
	^18^F-FMISO and ^18^F-FAZA	Hypoxia	Prognosis	Clinical evaluation
MRI/MRS	NA	Glx, NAA, choline	Diagnosis	Clinical evaluation
	^13^C-glucose	Glucose metabolism	Metabolic flux analysis, therapy response	Clinical evaluation
	^13^C-pyruvate			
	^2^H-labeled glucose/choline	Glucose/choline	Multi-metabolite mapping	Experimental modalities tested in animals
MSI	NA	Gemcitabine and its metabolites	Gemcitabine metabolism	Experimental modalities tested in animals
NIR imaging	V-1520	TSPO	Image-guided surgery	Experimental modalities tested in animals
	IRDye800CW	VEGFR2	Image-guided surgery	Clinical evaluation
Optical metabolic imaging	NA	NADPH and FAD	Therapy response	Experimental modalities tested in animals
US/PA/CEUS	NA	Oxygenation	Therapy response	Experimental modalities tested in animals
	VEGFR2-targeted microbubbles	VEGFR2	Diagnosis	Clinical evaluation

^1^ The stage of application in pancreatic cancer. PET: positron emission tomography; MRI: magnetic resonance imaging; MRS: magnetic resonance spectroscopy; MSI: mass spectrometry imaging; NIR: near-infrared; US: ultrasound; PA: photoacoustic; CEUS: contrast-enhanced ultrasound.

## Abbreviations

The following abbreviations are used in this manuscript:

^18^F-FAC	2′-deoxy-2′-^18^F-fluoro-β-d-arabinofuranosylcytosine
AI	Artificial intelligence
CAFs	Cancer-associated fibroblasts
CB2R	Cannabinoid receptor type 2
CD36	Fatty acid translocase
CECT	Contrast-enhanced computed tomography
CEUS	Contrast-enhanced ultrasound
Chkα	Choline kinase-α
dCK	Deoxycytidine kinase
dFdC	2′,2′-difluorodeoxycytidine
DMI	Deuterium metabolic imaging
FA	Fatty acid
FABPs	Fatty acid-binding proteins
FAD	Flavin adenine dinucleotide
FAPI	FAP inhibitor
FATPs	Fatty acid transport proteins
FCH	^18^F-fluoro-choline
FEC	^18^F-fluoroethyl-choline
FSPG	(4S)-4-(3-18F-Fluoropropyl)-l-glutamate
G6Pase	Glucose-6-phosphatase
Ga68	Gallium-68
Gln	Glutamine
GLUD1	Glutamate dehydrogenase
GLUL	Glutamate ammonia ligase
GLUT1	Glucose transporter1
GOT1	Aspartate transaminase
HK1/2	Hexokinase 1/2
HNSCC	Head and neck squamous cell carcinoma
HP	Hyperpolarized
IHC	Immunohistochemical
LDH	Lactate dehydrogenase
LDLR	Low-density lipoprotein receptor
MRI	Magnetic resonance imaging
MRS	Magnetic resonance spectroscopy
MSI	Mass spectrometry imaging
NAA	N-acetylaspartate
NADPH	Nicotinamide dinucleotide
NIR	Near-infrared
O_2_	Oxygen
OMI	Optical metabolic imaging
ORR	Optical redox ratio
OS	Overall survival
OXPHOS	Oxidative phosphorylation
PA	Photoacoustic
PC	Pancreatic cancer
PDAC	Pancreatic adenocarcinoma
PDGFRβ	Platelet-derived growth factor receptor beta
PDO	PDAC-derived patient organoids
PEGPH20	PEGylated recombinant human hyaluronidase
PET	Positron emission tomography
PFK1	Phosphofructokinase1
PFS	Progression-free survival
PPP	Pentose phosphate pathway
PSC	Pancreatic stellate cells
ROS	Reactive oxygen species
scRNAseq	Single-cell RNA sequencing
SUV	Standardized uptake value
TCA	Tricarboxylic acid
TME	Tumor microenvironment
TSPO	Translocator protein
US	Ultrasound
VEGFR2	Vascular endothelial growth factor receptor type 2
